# Determinants of Neonatal Mortality in the United States

**DOI:** 10.7759/cureus.43019

**Published:** 2023-08-06

**Authors:** Oluwasegun A Akinyemi, Mojisola E Fasokun, Terhas Asfiha Weldeslase, Deborah Makanjuola, Oluwafemi E Makanjuola, Ofure V Omokhodion

**Affiliations:** 1 Health Policy and Management, University of Maryland School of Public Health, College Park, USA; 2 Surgery, Howard University, Washington D.C., USA; 3 Epidemiology and Public Health, The University of Alabama at Birmingham, Birmingham, USA; 4 Surgery, Howard University College of Medicine, Washington D.C., USA; 5 Public Health, The University of Alabama, Birmingham, USA; 6 Medicine and Surgery, University of Ilorin, Ilorin, NGA; 7 Obstetrics and Gynecology, University College Hospital, Ibadan, NGA

**Keywords:** prenatal care, neonatal health outcomes, adverse pregnancy -fetal outcomes, risk factors, neonatal mortality

## Abstract

Introduction

Despite a notable reduction in infant mortality over recent decades, the United States, with a rate of 5.8 deaths per 1,000 live births, still ranks unfavorably compared to other developed countries. This improvement appears inadequate when contrasted with the country's healthcare spending, the highest among developed nations. A significant proportion of this infant mortality rate can be attributed to neonatal fatalities.

Objective

The present study aimed to determine the risk factors associated with neonatal deaths in the United States.

Method

Using the United States Vital Statistics records, we conducted a retrospective study on childbirths between 2015 and 2019 to identify risk factors for neonatal mortality. Our final multivariate analysis included maternal parameters like age, insurance type, education level, cesarean section rate, pregnancy inductions and augmentations, weight gain during pregnancy, birth weight, number of prenatal visits, pre-existing conditions like chronic hypertension and prediabetes, and pregnancy complications like gestational diabetes mellitus (GDM). These variables were incorporated to enhance our model's sensitivity and specificity.

Result

There were 51,174 neonatal mortalities. Mothers with augmentation of labor had a 25% reduction in neonatal mortalities (NM) (OR=0.75; 95% CI 0.72-0.79), while labor induction was associated with a 31% reduction in NM (OR=0.69; 95% CI 0.66-0.72). Women above 40 years had a 29% increase in NM rate (OR=1.29;95% CI 1.15-1.44). Women without prenatal care have a 22% increase in the risk of NM (OR=1.22; 95% CI 1.14-1.30). The present model has a 60.7% sensitivity and a 99.9% specificity.

Conclusion

In the present study, significant interventions such as labor induction, augmentation, and prenatal care were associated with improved neonatal outcomes. These findings could serve as an algorithm for improving neonatal outcomes in the United States.

## Introduction

Neonatal mortality (NM), defined as the death of a newborn within the first 28 days of life [1,2], continues to be a significant public health concern in the United States [3]. Despite the considerable advancements in neonatal care, the US continues to have a higher neonatal mortality rate compared to many other developed countries [4]. As of 2020, the neonatal mortality rate in the US was 3.4 per 1,000 live births, which is high compared to countries like Finland and Japan, with rates as low as 1.4 and 0.8, respectively [5,6]. These disparities highlight the urgent need to identify and address the contributing factors to neonatal mortality in the US.

Previous research has identified potential risk factors associated with neonatal mortality, including maternal health status and racial and socioeconomic variables [7-13]. However, the complex interplay of these factors and their relative contributions to neonatal mortality remains unclear. Understanding these associations is critical to guide policy interventions and improving neonatal survival rates.

There remains uncertainty and controversy regarding the associations between proxies of maternal and fetal outcomes, such as cesarean section, induction and augmentation of labor, and neonatal mortality [14-16]. Some studies have found that cesarean section is associated with decreased neonatal mortality, while others suggest no significant association [17]. Similarly, divergent findings exist regarding the impact of neonatal intensive care unit (NICU) admission, induction and augmentation of labor, and prenatal care on neonatal mortality. These discrepancies could be due to variations in study design, sample characteristics, statistical methods, and healthcare systems across studies. Given these inconsistencies, there is a clear need for further research to provide a more definitive understanding of these relationships within the US context.

This study addresses this gap by robustly examining these factors in a large, representative US sample. It provides valuable insights for clinicians, researchers, and policymakers in their quest to reduce neonatal mortality.

## Materials and methods

Study design and data sources

The data utilized for this investigation was meticulously extracted from the US Vital Statistics Records encompassing the years 2015-2019. As a comprehensive repository of birth and death records across the nation, the National Vital Statistics Records database provides unparalleled insight into the diverse patterns and influences shaping population health trends in the United States. This robust and widely-accepted source of data ensures a broad scope and enhances the reliability of our findings. The database's completeness and representativeness of the US population allow for a meticulous investigation into the subject matter of our study. Since the database is de-identified and publicly available, ethical clearance or Institutional Review Board approval was unnecessary.

Study population

We studied 19,417,305 deliveries recorded in the United States Vital Statistics records between January 2015 and December 2019. The study cohort included women of all races/ethnicities. However, women with missing vital information such as maternal race/ethnicity, BMI, age, and measured pregnancy outcomes were excluded from the study.

Patient characteristics and risk factors

This study identified covariates based on existing literature, including maternal, obstetric, and medical history. These factors included maternal race/ethnicity defined as non-Hispanic White (White), non-Hispanic Black (Black), Hispanic, non-Hispanic American Indian/ Alaska Native, non-Hispanic Asian/Pacific Islanders, non-Hispanic Mixed races, and non-Hispanic other (Other), maternal insurance types (private insurance, public insurance (Medicaid), and uninsured), and maternal education level defined as either high school, college, or advanced education. Other factors included in the study are maternal age, pre-pregnancy diabetes, hypertension, pre-pregnancy obesity, gestational diabetes mellitus (GDM), cesarean section and previous cesarean section (PCS), augmentation and labor induction, congenital malformations, and delivery weight.

Definition of study outcomes

The outcome of interest was neonatal death, defined as the death of an infant from delivery to the first 28 days of life.

Statistical analysis

Categorical variables in our study were represented as frequencies and percentages, while continuous variables were denoted as mean values with ± standard deviations. Comparative analysis for categorical and continuous variables was performed via Chi-squared and independent sample t-tests, respectively. A Chi-squared analysis allowed us to ascertain bivariate associations between the study variables and neonatal mortality.

For our final multivariate analysis, we included influential factors such as maternal age, race/ethnicity, education, insurance types, paternal age, race/ethnicity, and education level. In addition, we also factored in pre-pregnancy diabetes, chronic hypertension, pregnancy weight gain, PCS, labor induction, delivery weight, gestational age, and diverse classes of pre-pregnancy BMI (underweight, normal, obesity class I, II, and III). Before running the regression, we examined multicollinearity between the variables and found no significant issues. Additionally, we identified and addressed extreme outliers that could influence our data.

In cases where a significant association was found, we further performed a post hoc analysis across all groups to pinpoint the source of statistical significance. Using bivariate logistic models, the association of study variables with neonatal death was quantified by computing unadjusted odds ratios (ORs) and their corresponding 95% confidence intervals (CIs).

Our final multivariate analysis was adjusted at the delivery stage. We then established the association between the variables under consideration. The threshold for statistical significance was set at a two-tailed p-value<0.05. All statistical procedures were executed using STATA 16 (StataCorp, College Station, Texas).

## Results

Table 1 provides a detailed breakdown of the characteristics of the total population stratified by neonatal mortality status. Each of the key variables is examined for any association with neonatal mortality status. 

**Table 1:** Association between study variables and neonatal mortality (United States Vital Statistics Records 2015-2019)

Variables	Total population	Neonatal mortality	Alive	p-value
	(N=19,417,305)	(n=51,174)	(N=19,366,131)	
Race/ethnicity				<0.001
Non-Hispanic Whites	9,886,674 (51.4%)	18,541 (39.5%)	9,868,133 (51.5%)	
Non-Hispanic Blacks	2,741,989 (14.3%)	13,520 (28.8%)	2,728,469 (14.2%)	
Hispanic	4,608,307 (24.0%)	10,144 (21.6%)	4,598,163 (24.0%)	
Non-Hispanic American Indian/ Alaskan Native	148,216 (0.8%)	396 (0.8%)	147,820 (0.8%)	
Non-Hispanic Asian/ Pacific Islanders	1,220,184 (6.4%)	2,147 (4.6%)	1,218,037 (6.4%)	
Non-Hispanic Mixed Races	57,601 (0.3%)	198 (0.4%)	57,403 (0.3%)	
Others	404,034 (2.1%)	958 (2.0%)	403,076 (2.1%)	
Unknown	159,552 (0.8%)	1,060 (2.3%)	158,492 (0.8%)	
Age (years)				<0.001
≤ 19	999,242 (5.2%)	3,224 (6.9%)	995,077 (5.2%)	
20-24	3,849,333 (20.0%)	9,961 (21.2%)	3,836,253 (20.0%)	
25-29	5,579,992 (29.0%)	13,211 (28.1%)	5,562,494 (29.0%)	
30-34	5,444,285 (28.3%)	11,792 (25.1%)	5,427,849 (28.3%)	
35-39	2,751,078 (14.3%)	6,740 (14.4%)	2,741,290 (14.3%)	
≥40	619,673 (3.2%)	2,036 (4.3%)	616,630 (3.2%)	
Education				<0.001
Elementary	2,521,226 (13.3%)	7,172 (16.3%)	2,511,648 (13.3%)	
Pre-college	8,730,947 (45.9%)	23,582 (53.4%)	8,698,588 (45.9%)	
Tertiary	5,469,446 (28.8%)	10,042 (22.8%)	5,455,712 (28.8%)	
Postgraduate	2,284,856 (12.0%)	3,343 (7.6%)	2,280,190 (12.0%)	
Insurance				<0.001
Medicaid	8,155,056 (42.6%)	22,643 (48.8%)	8,123,043 (42.6%)	
Private	9,430,667 (49.3%)	19,526 (42.1%)	9,403,953 (49.3%)	
Self-pay	810,031 (4.2%)	2,516 (5.4%)	807,532 (4.2%)	
Other	734,066 (3.8%)	1,746 (3.8%)	731,574 (3.8%)	
BMI				< 0.001
Underweight	631,751 (3.4%)	1,393 (3.2%)	629,782 (3.4%)	
Normal	8,112,616 (43.2%)	14,793 (34.3%)	8,091,332 (43.2%)	
Overweight	4,944,021 (26.3%)	10,793 (25.1%)	4,928,556 (26.3%)	
Obese class I	2,765,555 (14.7%)	7,757 (18.0%)	2,754,815 (14.7%)	
Obese class II	1,356,161 (7.2%)	4,530 (10.5%)	1,350,082 (7.2%)	
Obese class III	964,854 (5.1%)	3,819 (8.9%)	959,818 (5.1%)	
Weight gain (pounds)				<0.001
< 11	1,789,968 (9.6%)	16,956 (40.4%)	1,770,873 (9.5%)	
11-20	3,238,566 (17.4%)	11,706 (27.9%)	3,222,842 (17.4%)	
21-30	5,275,252 (28.3%)	7,077 (16.9%)	5,263,584 (28.4%)	
31-40	4,515,185 (24.3%)	3,490 (8.3%)	4,508,153 (24.3%)	
≥ 41	3,803,019 (20.4%)	2,702 (6.4%)	3,796,842 (20.5%)	
Birth weight (g)				<0.001
<2500	1,569,431 (8.2%)	37,608 (88.1%)	1,523,116 (7.9%)	
2500-3999	16,168,597 (84.1%)	4,720 (11.1%)	16,156,951 (84.3%)	
4000-4499	1,292,826 (6.7%)	263 (0.6%)	1,291,752 (6.7%)	
≥4500	200,845 (1.0%)	123 (0.3%)	200,295 (1.0%)	
Prediabetes	173,297 (0.9%)	978 (2.1%)	171,770 (0.9%)	<0.001
Chronic hypertension	366,503 (1.9%)	1,772 (3.8%)	364,085 (1.9%)	<0.001
Previous cesarean section	2,978,278 (15.5%)	6,262 (13.4%)	2,966,612 (15.5%)	<0.001
Breastfeeding	13,769,505 (82.2%)	2,598 (7.2%)	13,766,907 (82.3%)	<0.001
Labor induction	5,010,014 (26.1%)	6,124 (13.1%)	5,003,890 (26.1%)	<0.001
Labor augmentation	4,093,612 (21.3%)	4,211 (9.0%)	4,089,401 (21.3%)	<0.001
Duration of prenatal care				<0.001
1-3 months	14,486,951 (77.4%)	30,231 (71.9%)	14,456,720 (77.4%)	
4-6 months	3,089,336 (16.5%)	6,707 (16.0%)	3,082,629 (16.5%)	
7-final months	847,783 (4.5%)	764 (1.8%)	847,019 (4.5%)	
No prenatal care	305,830 (1.6%)	4,348 (10.3%)	301,482 (1.6%)	

The majority of the total population are Non-Hispanic Whites (NHW; 51.4%), followed by Hispanics (24.0%), Non-Hispanic Blacks (NHB; 14.3%), Non-Hispanic American Indian/ Alaskan Native (NHAIAN; 0.8%), Non-Hispanic Asian/Pacific Islander (NHAPI); 6.4%), Non-Hispanic Mixed Races (NHMR); 0.3%), Others (2.1%) and Unknown (0.8%). The proportion of neonatal deaths was highest among NHWs (39.5%), followed by NHBs (28.8%) and Hispanics (21.6%).

Most of the population were aged 25-29 (29%) and 30-34 (28.3%). Neonatal deaths were most prevalent in the age groups 25-29 (28.1%). Most of the population had pre-college education (45.9%), followed by tertiary education (28.8%). Among the neonatal deaths, most were from mothers with pre-college education (53.4%).

The population predominantly utilized private insurance (49.3%) or Medicaid (42.6%). Most neonatal deaths occurred among those covered by Medicaid (48.8%). Most of the population had a normal BMI (43.2%) or were overweight (26.3%). Most neonatal deaths occurred among obese women (37.4%). Most of the population gained 21-30 lbs (28.3%) during pregnancy. Most neonatal deaths occurred among those who gained less than 11 lbs during pregnancy (40.4%).

Most of the population had a birth weight between 2.5-4.0Kg grams (84.1%). Most neonatal deaths occurred among those with a birth weight of less than 2.5kg grams (88.1%).

Prediabetes, chronic hypertension, previous cesarean section, breastfeeding, labor induction, labor augmentation, and prenatal care visits also show statistically significant differences between the neonates who experienced neonatal death and those who survived.

The presence of statistically significant differences in each characteristic between neonates who passed away and those who survived indicates that these factors may be associated with neonatal mortality. However, this table alone does not provide specific relationships or causal links between these characteristics and neonatal mortality. 

Multivariate analysis

Table 2 highlights the influence of congenital malformations on the risk of neonatal mortality. As expected, conditions such as anencephaly, omphalocele, meningomyelocele, congenital heart disease (CHD), and hernia significantly increased the odds of neonatal mortality with odds ratios of 46.79, 6.59, 4.55, 20.82, and 18.38, respectively, all of which were statistically significant (p-value<0.05).

**Table 2:** Congenital abnormalities and risk of neonatal mortality (United States Vital Statistics 2015-2019)

Neonatal mortality	Odds ratio	Lower CI	Upper CI	p-value
Anencephaly	46.79	38.05	57.54	<0.001
Omphalocele	6.59	4.99	8.7	<0.001
Meningomyelocele	4.55	3.15	6.56	<0.001
Congenital heart disease	20.82	18.18	23.84	<0.001
Congenital hernia	18.38	14.77	22.87	<0.001

Table 3 shows other significant predictors of neonatal deaths in the present study. Pre-existing maternal conditions such as prediabetes increased the odds of neonatal mortality (OR=1.33, p-value<0.05), while pre-hypertension decreased the odds (OR=0.90, p-value<0.05). GDM was associated with lower odds of neonatal mortality (OR=0.70, p-value<0.05).

**Table 3:** Predictors of neonatal mortality (United States Vital Statistics Records 2015-2019) GDM - gestational diabetes mellitus

Variables	Odds ratio	Lower CI	Upper CI	p-value
Maternal age				
≤ 19	Reference	Reference	Reference	
20-24	1	0.93	1.07	0.921
25-29	1.07	1	1.14	0.054
30-34	1.04	0.97	1.12	0.276
35-39	1.07	0.99	1.15	0.1
≥40	1.27	1.15	1.4	<0.001
Race/ethnicity				
Non-Hispanic Whites	Reference	Reference	Reference	
Non-Hispanic Blacks	0.83	0.79	0.86	<0.001
Hispanic	1.03	0.98	1.07	0.259
Non-Hispanic American Indian/ Alaska Native	0.91	0.77	1.07	0.24
Non-Hispanic Asian/Pacific Islander	0.9	0.84	0.97	0.005
Non-Hispanic Other	1.13	0.88	1.45	0.33
Non-Hispanic Mixed Races	0.88	0.79	0.97	0.014
Unknown	1.2	0.95	1.51	0.121
Education				
Elementary	Reference	Reference	Reference	
Pre-college	1.04	1	1.09	0.074
Tertiary	1.07	1.01	1.33	0.018
Postgraduate	1.03	0.96	1.11	0.37
Insurance				
Medicaid	Reference	Reference	Reference	
Private	1.13	1.09	1.17	<0.001
Self-Pay	1.13	1.05	1.21	0.002
Other	0.95	0.88	1.03	0.186
Body mass index (Kg/M2)				
Normal	Reference	Reference	Reference	
Underweight	0.89	0.82	0.97	0.008
Overweight	1.06	1.02	1.11	0.002
Obese class I	1.09	1.04	1.14	<0.001
Obese class II	1.1	1.04	1.16	0.001
Obese class III	1.08	1.01	1.47	0.016
Weight gain (grams)				
< 11	Reference	Reference	Reference	
11-20	0.74	0.71	0.77	<0.001
21-30	0.53	0.51	0.55	<0.001
31-40	0.44	0.42	0.47	<0.001
≥ 41	0.46	0.43	0.48	<0.001
No prenatal care	1.22	1.14	1.31	<0.001
Birth weight (grams)				
2500-3999	Reference	Reference	Reference	
4000-4499	0.68	0.59	0.78	<0.001
≥4500	1.49	1.21	1.83	<0.001
Chronic hypertension	0.9	0.83	0.97	<0.001
Prediabetes	1.33	1.2	1.48	< 0.001
GDM	0.7	0.65	0.75	<0.001

Insurance type and mother's race showed mixed results. Private insurance and self-pay were associated with slightly higher odds of neonatal mortality (OR=1.13 for both, p-value<0.001). In contrast, other types of insurance did not significantly affect the odds (p-value>0.05). Non-Hispanic Blacks have 17% lower odds of neonatal mortalities than non-Hispanic whites. 

Mother's education did not show a clear pattern, with pre-college and postgraduate education showing no significant association with neonatal mortality. However, tertiary education had slightly higher odds (OR=1.07, p-value<0.05). Women aged 40 years or older had higher odds of neonatal mortality (OR=1.27, p-value<0.05). Increasing BMI categories were associated with increased odds of neonatal mortality. The lack of prenatal care was also associated with higher odds of neonatal mortality (OR=1.22, p-value<0.05).

**Figure 1:**
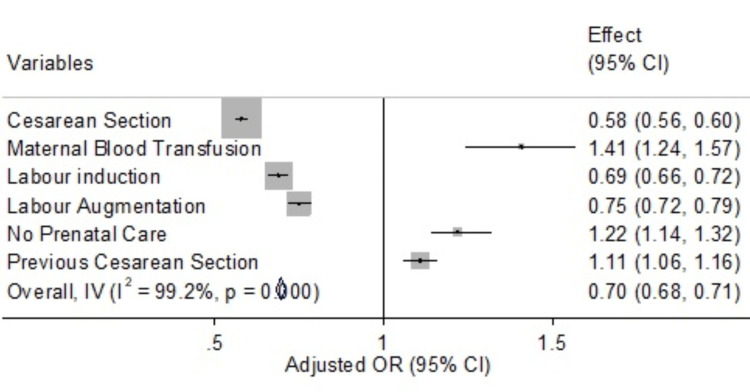
Selected predictors of neonatal mortality (United States Vital Statistics Records 2015-2019)

Figure 1 highlights the association between selected maternal and neonatal outcome measures and neonatal mortality. Maternal and neonatal outcomes, such as cesarean delivery and maternal blood transfusions, are associated with a significant risk of neonatal death. Labor conditions such as labor induction and augmentation tend to be protective against neonatal mortality. Cesarean delivery was associated with reduced odds of neonatal mortality (OR=0.58, p-value<0.001). This could be because cesarean deliveries are often performed where a vaginal delivery might pose a risk to the mother or baby. Therefore, a cesarean delivery could potentially prevent complications that might lead to neonatal mortality.

Induction of labor was associated with decreased odds of neonatal mortality (OR=0.69, p-value<0.001). Labor induction can be planned for various reasons, such as the baby being overdue or distressed or the mother having health problems. This early intervention can potentially mitigate risks, leading to a decreased likelihood of neonatal mortality.

Augmentation, which is the process of stimulating the uterus to increase the frequency, duration, and intensity of contractions after labor has started spontaneously, was also associated with decreased odds of neonatal mortality (OR=0.75, p-value<0.05).

## Discussion

In the present study, we explored the association between several baseline characteristics, preexisting comorbidities, and labor conditions and how these factors influence neonatal mortality in the United States.

Regarding mode of delivery, our results reveal that cesarean delivery is significantly associated with lower neonatal mortality odds (OR=0.58, p-value<0.05). This result might be more applicable to preterm neonates and macrosomic babies, aligning with prior studies [18,19]. Cesarean deliveries are often performed when vaginal delivery might present risks to the mother or infant, which could explain the decreased neonatal mortality observed [20]. However, some studies report contrary findings, suggesting higher neonatal mortality rates associated with cesarean sections [19,20]. This discrepancy could be due to the varying criteria for choosing a cesarean section across different settings, potential complications from surgery, or differences in postoperative care. Our results imply that careful patient selection for cesarean delivery could be instrumental in neonatal mortality reduction strategies.

Our results suggest that labor induction is associated with decreased neonatal mortality odds (OR=0.69, p-value<0.05). Labor induction can mitigate risks associated with overdue or distressed babies or maternal health issues. Previous studies corroborate this finding, and the mother's age and gestational age are significant factors [21]. Our results highlight the need for appropriate candidate selection for labor induction to improve neonatal survival rates.

In addition, our findings show that labor augmentation is associated with decreased neonatal mortality odds (OR=0.75, p-value<0.05). However, to minimize neonatal adverse outcomes, augmentation should be monitored carefully with a standardized treatment regimen [22]. Our findings could have significant policy implications, pointing to the need for guidelines on labor augmentation to minimize neonatal mortality.

In the present study, conditions such as anencephaly, omphalocele, meningomyelocele, congenital heart disease (CHD), and congenital hernia were significantly associated with increased odds of neonatal mortality, aligning with existing literature that identifies these conditions as significant risk factors for neonatal mortality [23-27]. These associations underscore the necessity of early detection and intervention, particularly for CHD and meningomyelocele, where appropriate prenatal and postnatal management could improve survival rates [27-29]. Our results suggest that healthcare policies should prioritize prenatal screening protocols to facilitate early diagnosis and management of these conditions.

Maternal preexisting conditions were found to impact neonatal mortality odds significantly. Prediabetes increased the odds of neonatal mortality (OR=1.33, p-value<0.05), consistent with previous studies that linked impaired glucose tolerance to adverse neonatal outcomes [30,31]. Conversely, our study found that pre-hypertension was associated with decreased odds of neonatal mortality (OR=0.90, p-value<0.05). This contradicts some studies which reported increased neonatal risks associated with hypertensive disorders [32]. The differing findings may arise from differences in study populations, diagnostic criteria, or management of hypertension during pregnancy. Interestingly, we also found that GDM was associated with lower neonatal mortality odds (OR=0.70, p-value<0.05), possibly related to increased surveillance and intervention in pregnancies affected by GDM. These findings suggest the need for comprehensive maternal care protocols to manage preexisting and pregnancy-related conditions.

We observed that insurance type and maternal race had varying associations with neonatal mortality. Private insurance and self-pay were associated with slightly higher mortality odds, contrasting with studies suggesting that better insurance coverage may improve neonatal outcomes [33,34]. This disparity could stem from differential access to care, variability in the quality of care, or underlying social determinants of health not captured in our study. Non-Hispanic Blacks, non-Hispanic Asian/Pacific Islanders, and non-Hispanic Mixed Race mothers had lower odds of neonatal mortality. This finding partially contradicts existing literature which often reports higher neonatal mortality rates among non-Hispanic Black populations [34]. Such discrepancies highlight the complexity of racial disparities in healthcare outcomes, warranting further research into the specific drivers of these variations.

Our results also showed that maternal education was not consistently associated with neonatal mortality, with only tertiary education associated with slightly higher odds. This may reflect the mixed findings in the existing literature on the relationship between maternal education and neonatal outcomes [35]. Variations in the definition of education levels and cultural and geographical differences in educational access and quality may contribute to these inconsistencies.

The mother's age was found to impact neonatal mortality, with mothers aged 40 or older having higher odds (OR=1.27, p-value<0.05). This is consistent with prior studies highlighting advanced maternal age as a risk factor for neonatal mortality [36,37]. Furthermore, we found a significant association between obesity and neonatal mortality rates, with the higher BMI group (overweight and obese groups) having a higher neonatal mortality rate than women with normal BMI, corroborating existing literature [38,39]. These findings underline the importance of preconception and antenatal care strategies focusing on healthy weight management to reduce neonatal mortality.

Lastly, lack of prenatal care was associated with increased neonatal mortality odds, echoing prior findings on the importance of prenatal care in ensuring optimal neonatal outcomes [40]. This underscores the need for adequate health policies guaranteeing universal access to high-quality prenatal care.

Policy implication and public health significance

This study underscores the substantial influence of neonatal conditions, maternal health, and socioeconomic factors on neonatal mortality, with broad policy implications and public health significance. Emphasizing early detection and management of neonatal conditions such as anencephaly, CHD, and meningomyelocele, policymakers should enhance prenatal screening protocols and access to specialized neonatal care.

Maternal health concerns, such as prediabetes and gestational diabetes, highlight the necessity for comprehensive preconception and antenatal care. Policy initiatives should focus on managing preexisting conditions, weight management, and encouraging optimal gestational weight gain. The decreased odds of neonatal mortality with gestational diabetes may reflect effective care and monitoring protocols for these mothers, providing a model for other risk conditions.

The mixed findings on insurance type and racial disparities indicate the need to address systemic health inequities. Policies should ensure universal access to high-quality prenatal care and reduce differences in neonatal outcomes.

These findings highlight the need for comprehensive, multi-level strategies to reduce neonatal mortality, informing the development of evidence-based policies and interventions tailored to individual and population-level risk profiles.

Limitations and strengths

This study's strengths lie in its comprehensive examination of various factors associated with neonatal mortality, providing a holistic understanding of potential risk factors. The large sample size enhances the power of the study, providing robust evidence for the associations identified. Moreover, the multivariable analysis allows us to control for potential confounding factors, improving the validity of our findings.

Despite these strengths, the study has several limitations. First, being an observational study, it is subject to potential residual confounding from unmeasured factors. We also acknowledge the potential for information bias, as data depends on the accuracy of medical records, which might vary across different settings.

Despite finding significant associations, the study's cross-sectional design prevents us from inferring causality. Additionally, there might be variations in diagnostic criteria and care practices across different regions and institutions, which our study could not account for.

While the study is broadly representative, the generalizability of the findings may be limited due to potential differences in healthcare access, quality of care, and social determinants of health in different geographical locations.

Finally, the study did not capture long-term neonatal outcomes, and future research could focus on understanding how these factors influence long-term survival and morbidity.

Despite these limitations, our study provides valuable insights into the complex interplay of factors influencing neonatal mortality, aiding in better-informed healthcare decisions and policy-making.

## Conclusions

Our study demonstrated significant associations between neonatal mortality and a multitude of factors, including neonatal conditions, maternal pre-existing and gestational conditions, socio-economic indicators, and maternal age and BMI. Our findings underline the importance of early detection of neonatal conditions, management of maternal health, and the need to address systemic health disparities to mitigate neonatal mortality. Although the associations identified are observational and warrant further investigation, they underscore the potential for policy interventions targeting these factors to improve neonatal survival rates. Future research should focus on understanding the mechanisms underlying these associations and developing effective interventions tailored to individual risk profiles. Despite the limitations, our study provides valuable insights that can inform policy decisions and contribute to improved neonatal health outcomes.
